# Supplementary material for: Behavioral factors and SARS-CoV-2 transmission heterogeneity within a household cohort in Costa Rica
Source: Commun Med (Lond). 2023 Jul 22;3:102. doi: 10.1038/s43856-023-00325-6 (PMC10363136; doi:10.1038/s43856-023-00325-6)
Supplement: Supplementary file 1 — Supplementary Information [file 43856_2023_325_MOESM1_ESM.pdf]

Supplementary information:

Behavioral factors and SARS-CoV-2 transmission heterogeneity within a household cohort in Costa Rica

Kaiyuan Sun[1], Viviana Loria[2], Amada Aparicio[3], Carolina Porras[2], Juan Carlos Vanegas[2], Michael Zúñiga[2], Melvin Morera[3], Carlos Avila[2], Arturo Abdelnour[3], Mitchell H. Gail[4], Ruth Pfeiffer[4], Jeffrey I. Cohen[5], Peter D. Burbelo[6], Mehdi A Abed[6], Cécile Viboud[1], Allan Hildesheim[4], Rolando Herrero[2], D Rebecca Prevots[7], for the RESPIRA Study Group[8]

[1]Division of International Epidemiology and Population Studies, Fogarty International Center, National Institutes of Health (NIH), Bethesda, MD

[2]Agencia Costarricense de Investigaciones Biomédicas (ACIB) – Fundación INCIENSA (FUNIN) , San Jose, Costa Rica

[3]Caja Costaricense de Seguro Social, San Jose, Costa Rica

[4]Division of Cancer Epidemiology and Genetics, National Cancer Institute, NIH, Bethesda, MD

[5]Laboratory of Infectious Diseases, National Institute of Allergy and Infectious Diseases (NIAID), NIH, Bethesda, MD

[6]National Institute of Dental and Craniofacial Research, NIH, Bethesda, MD

[7]Epidemiology and Population Studies Unit, Laboratory of Clinical Immunology and Microbiology, Division of Intramural Research, NIAID, NIH, Bethesda, MD

[8]RESPIRA Study Group: Caja Costariccense de Seguro Social: Amada Aparicio, Karla Moreno, Roy Wong, Melvin Morera, Arturo Abdelnour, Alejandro Calderon, Kattia Camacho, Gabriela Ivankovich, Adriana Yock ; Ministerio de Salud, Costa Rica: Roberto Castro. Agencia Costarricense de Investigaciones Biomédicas (ACIB) – Fundación INCIENSA (FUNIN): . Rolando Herrero, Carolina Porras, Bernal Cortés, Viviana Loría, Rebecca Ocampo, Michael : Zúñiga; Universidad de Costa Rica (UCR) ; Cristina Barboza-Solis, Romain Fantin : Universidad de Costa Rica

Correspondence to:

D Rebecca Prevots, Epidemiology and Population Studies Unit, Laboratory of Clinical Immunology and Microbiology, Division of Intramural Research, NIAID, NIH, Bethesda, MD

rprevots@niaid.nih.gov

or

Kaiyuan Sun, Division of International Epidemiology and Population Studies, Fogarty International Center, National Institutes of Health (NIH), Bethesda, MD

kaiyuan.sun@nih.gov

Table S1: Model selection on the risk factors for household chain-binomial transmission model.

	**Model**	**0**	**1**	**2**	**3**	**4**	**5**	**6**	**7**	**8**	**9**	**10**	**11**	**12**	**13**	**14**	**15**
	**Log Likelihood**	477	476	475	467	467	465	463	462	437	429	420	418	416	415	413	412
	**Akaike Information Criterion (AIC)**	959	955	965	957	957	957	955	954	907	891	876	876	876	877	878	880
	**ΔAIC**	0	-4	+6	-2	-2	-2	-4	-5	-52	-68	-83	-83	-83	-82	-81	-79
**Baseline**	Baseline community risk	*	*	*	*	*	*	*	*	*	*	*	*	*	*	*	*
	Index case & seropositive household members	*															
	Index case		*	*	*	*	*	*	*	*	*	*	*	*	*	*	*
	Seropositive household members		*	*	*	*	*	*	*	*	*	*	*	*	*	*	*
**Risk factors**	Cumulative incidence rate (relative community risk)		*	*	*	*	*	*	*	*	*	*	*	*	*	*	*
	Age, transmissibility			*	*	*	*	*	*	*	*	*	*	*	*	*	*
	Age, susceptibility				*	*	*	*	*	*	*	*	*	*	*	*	*
	Sex, transmissibility					*	*	*	*	*	*	*	*	*	*	*	*
	Sex, susceptibility						*	*	*	*	*	*	*	*	*	*	*
	Obesity, transmissibility							*	*	*	*	*	*	*	*	*	*
	Obesity, susceptibility								*	*	*	*	*	*	*	*	*
	Household size									*							
	Log2 (No. of household contacts)										*	*	*	*	*	*	*
	Shared bedroom with index (Y/N)											*	*	*	*	*	*
	Cared for index (Y/N)												*	*	*	*	*
	If wore mask > half of the time (index case)													*	*	*	*
	If wore mask > half of the time (household member)														*	*	*
	Hours with index outside bedroom															*	*
	If symptomatic																*

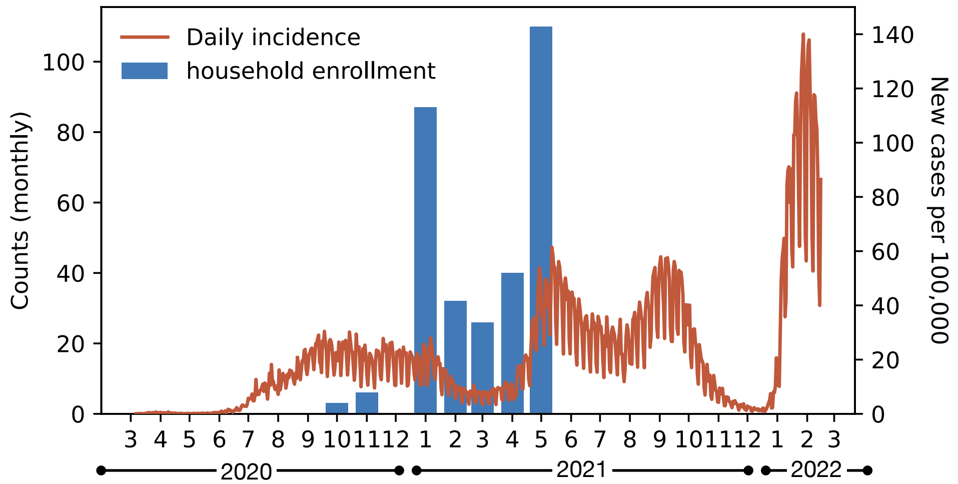

Figure S1: Daily incidence of reported SARS-CoV-2 cases in Costa Rica (red line) and the monthly number of enrolled households based on the index cases' diagnosis time (blue bar).

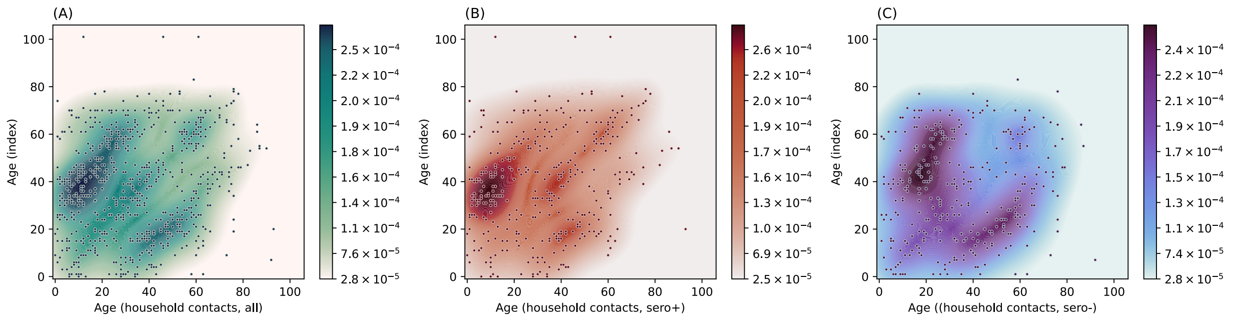

Figure S2: (A) Contact patterns between index cases and household members. Each dot represents the age pairs between index and household member and heatmap represents the frequency. (B) Same as (A) but only for seropositive household members only. (C) Same as (A) but for seronegative household members only. Colorbars indicate probability density.

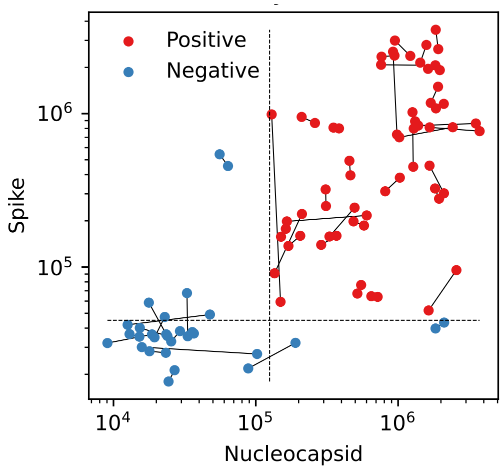

Figure S3: The results of the serologic readout for the quality control samples (including both pre-pandemic samples as negative control and a subset of the study sample). Each sample were tested in duplicate (connected by the solid line). The dashed lines are pre-defined positive cutoff for both the Nucleocapsid and the Spike protein. We require a seropositive sample (red dots) as being positive on both spike and nucleocapsid proteins and seropositive otherwise (blue dots). For the quality control samples, all duplicates give consistent results on seropositivity.

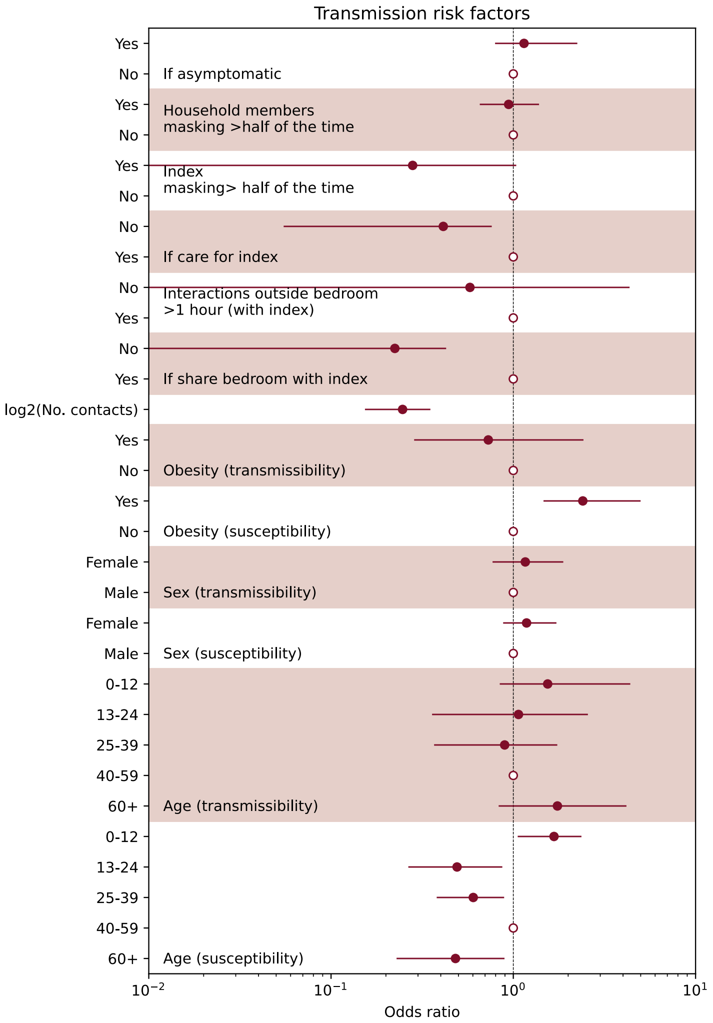

Figure S4: Estimates from the chain-binomial household transmission model with household level bootstrapping. Solid dots and horizontal lines represent point estimates and 95% confidence intervals of the adjusted odds ratios of transmission risk factors. Circles represent the reference class.

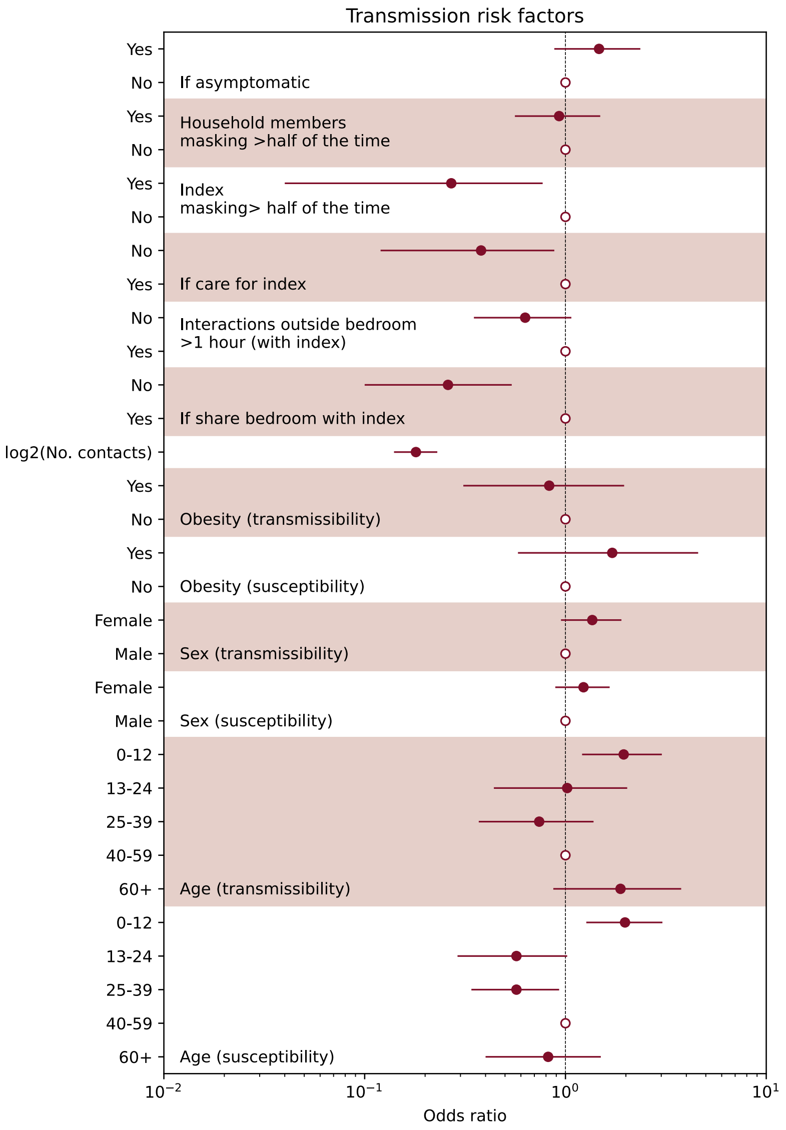

Figure S5: Estimates from the chain-binomial household transmission model excluding households with either index case or household members who had received vaccination prior or during the study period. Solid dots and horizontal lines represent point estimates and 95% confidence intervals of the adjusted odds ratios of transmission risk factors. Circles represent the reference class.
