# Supplementary material for: Behavioral factors and SARS-CoV-2 transmission heterogeneity within a household cohort in Costa Rica
Source: Commun Med (Lond). 2023 Jul 22;3:102. doi: 10.1038/s43856-023-00325-6 (PMC10363136; doi:10.1038/s43856-023-00325-6)
Supplement: Supplementary file 2 — Reporting Summary [file 43856_2023_325_MOESM2_ESM.pdf]

nature portfolio

Corresponding author(s): Kaiyuan Sun, Rebecca Prevots

Last updated by author(s): Feb 14, 2023

Reporting Summary

Nature Portfolio wishes to improve the reproducibility of the work that we publish. This form provides structure for consistency and transparency in reporting. For further information on Nature Portfolio policies, see our Editorial Policies and the Editorial Policy Checklist.

Statistics

For all statistical analyses, confirm that the following items are present in the figure legend, table legend, main text, or Methods section.

n/a	Confirmed	
☐	☒	The exact sample size (*n*) for each experimental group/condition, given as a discrete number and unit of measurement
☐	☒	A statement on whether measurements were taken from distinct samples or whether the same sample was measured repeatedly
☐	☒	The statistical test(s) used AND whether they are one- or two-sided<br>*Only common tests should be described solely by name; describe more complex techniques in the Methods section.*
☐	☒	A description of all covariates tested
☒	☐	A description of any assumptions or corrections, such as tests of normality and adjustment for multiple comparisons
☐	☒	A full description of the statistical parameters including central tendency (e.g. means) or other basic estimates (e.g. regression coefficient) AND variation (e.g. standard deviation) or associated estimates of uncertainty (e.g. confidence intervals)
☒	☐	For null hypothesis testing, the test statistic (e.g. F, t, r) with confidence intervals, effect sizes, degrees of freedom and P value noted<br>*Give P values as exact values whenever suitable.*
☒	☐	For Bayesian analysis, information on the choice of priors and Markov chain Monte Carlo settings
☒	☐	For hierarchical and complex designs, identification of the appropriate level for tests and full reporting of outcomes
☒	☐	Estimates of effect sizes (e.g. Cohen's *d*, Pearson's *r*), indicating how they were calculated

Our web collection on statistics for biologists contains articles on many of the points above.

Software and code

Policy information about availability of computer code

Data collection	Data were entered during visits on tablet computers with use of the Research Electronic Data Capture application (REDCap).
Data analysis	Software packages used: python version 3.8.11; scipy version 1.7.1.

For manuscripts utilizing custom algorithms or software that are central to the research but not yet described in published literature, software must be made available to editors and reviewers. We strongly encourage code deposition in a community repository (e.g. GitHub). See the Nature Portfolio guidelines for submitting code & software for further information.

Data

Policy information about availability of data

All manuscripts must include a data availability statement. This statement should provide the following information, where applicable:

- Accession codes, unique identifiers, or web links for publicly available datasets
- A description of any restrictions on data availability
- For clinical datasets or third party data, please ensure that the statement adheres to our policy

The investigators welcome inquiries about possible collaborations and requests for access to the dataset. Anonymized datasets as required can be shared after approval of a proposal and a signed data use agreement. Investigators interested in more details about this study, including protocols and informed consent forms should contact the principal investigator and corresponding author, D Rebecca Prevots (rprevots@niaid.nih.gov). Aggregate data to reproduce the figures are available at https://doi.org/10.5281/zenodo.779379526

Human research participants

Policy information about studies involving human research participants and Sex and Gender in Research.

Reporting on sex and gender	Information on sex were collected based on self-reporting.
Population characteristics	Participant characteristics are summarized in Table 1 of the manuscript.
Recruitment	For the larger prospective study, 1000 cases were recruited from three geographic areas: Puntarenas Province, Greater San Jose Metropolitan Area - (Gran Area Metropolitano), and the province of Guanacaste, and four age strata (0-19, 20-39, 40-59, 60+) using national surveillance lists provided by the CCSS and Health Ministry. Only PCR confirmed cases were included in the lists surveillance system, because they met the case definition used for surveillance. The geographic areas were selected based on logistic considerations and represented 58% percent of the Costa Rican population. Cases were sampled randomly within each geographic area and age stratum. . Approximately 30% of cases were approached for consent to participate in the nested household study; these cases were termed "index" cases.<br>A household was defined as two or more people living together who shared a kitchen. To be eligible for inclusion, a contact must have spent at least one night per week in the living area since the diagnosis of the index case. After consent and enrollment, index cases and their household contacts were administered a questionnaire to ascertain demographic, clinical, and behavioral risk and preventive factors. For household contacts, symptoms related to SARS-CoV-2 were ascertained for the time period two weeks before or two weeks after the sample collection date for the index case (referred to hereafter as "date of diagnosis"). If a household contact reported a prior diagnosis of COVID, symptoms were ascertained in relation to that diagnosis. Blood samples were collected from household contacts 30 to 60 days after the date of collection of the PCR-confirmed positive sample of the index case, and serum samples were tested to ascertain the presence of SARS-CoV-2 antibodies (against both SARS-CoV-2 nucleocapsid and spike protein), as a marker of past SARS-CoV-2 infection.<br>Household index cases and their contacts were enrolled from December 1, 2020, through July 31, 2021. This period coincided with the middle of the first wave and the end of the second wave in Costa Rica (Figure S1). The study was conducted immediately prior to the widespread availability of SARS-CoV-2 vaccines in Costa Rica21. Once study recruitment had been completed, national vaccination registries were searched to ascertain vaccination status and dates for any participants who had been vaccinated.
Ethics oversight	The RESPIRA study protocol was approved by the Central Institutional Review Board of the CCSS. (Protocol R020-SABI-000261). Informed, signed consent was obtained from all study participants or their proxies.

Note that full information on the approval of the study protocol must also be provided in the manuscript.

Field-specific reporting

Please select the one below that is the best fit for your research. If you are not sure, read the appropriate sections before making your selection.

☒ Life sciences ☐ Behavioural & social sciences ☐ Ecological, evolutionary & environmental sciences

For a reference copy of the document with all sections, see nature.com/documents/nr-reporting-summary-flat.pdf

Life sciences study design

All studies must disclose on these points even when the disclosure is negative.

Sample size	The sample size selected was a function of both logistic considerations as well as anticipated power. During the design phase of the study in the spring of 2020, we consulted with existing guidance from the World Health Organization (WHO) on statistical considerations for design of sero-epidemiologic studies for SARS-CoV-2 (https://apps.who.int/iris/handle/10665/331656) , and also reviewed published studies on household transmission to better understand the potential range of parameter estimates and our power to detect these. Based on available knowledge at the time, we hypothesized that a sample of 200 would give us a reasonable power to estimate secondary transmission and risk factors. Given all these considerations, we obtained intramural funding to recruit 300 households, and this number was exceeded slightly, for a total of 304 households.
Data exclusions	For the main analysis of the study, no data were excluded. We conducted sensitivity analysis excluding household with vaccinated participants, this is clearly stated in the manuscript.
Replication	NA (This is a prospective household observational study during the COVID-19 pandemic, circulating variants and population immunity have fundamentally changed throughout the study thus it is impossible to replicate the real-world epidemiological situations in a similar population)
Randomization	NA
Blinding	NA (This is a prospective household observational study, no blinding performed)

Reporting for specific materials, systems and methods

We require information from authors about some types of materials, experimental systems and methods used in many studies. Here, indicate whether each material, system or method listed is relevant to your study. If you are not sure if a list item applies to your research, read the appropriate section before selecting a response.

Materials & experimental systems

n/a	Involved in the study
☒	☐ Antibodies
☒	☐ Eukaryotic cell lines
☒	☐ Palaeontology and archaeology
☒	☐ Animals and other organisms
☐	☒ Clinical data
☒	☐ Dual use research of concern

Methods

n/a	Involved in the study
☒	☐ ChIP-seq
☒	☐ Flow cytometry
☒	☐ MRI-based neuroimaging

Clinical data

Policy information about clinical studies

All manuscripts should comply with the ICMJE guidelines for publication of clinical research and a completed CONSORT checklist must be included with all submissions.

Clinical trial registration	NCT04537338
Study protocol	*Note where the full trial protocol can be accessed OR if not available, explain why.*
Data collection	*Describe the settings and locales of data collection, noting the time periods of recruitment and data collection.*
Outcomes	*Describe how you pre-defined primary and secondary outcome measures and how you assessed these measures.*
